# The Late Meeting at Long Branch

**Published:** 1875-09

**Authors:** 


### EDITORIAL. ETC.

---

*The Late Meeting at Long Branch.*—The following account under the heading of "Dentists to the Front"—"A Poet"—"Fun among the Dental fraternity at Long Branch"—"Buying up the Press"—appears in the *New York Evening Express* of August 12th, 1875. Who the parties referred to are, we do not know, as the proceedings have not yet been published, but the person feeling most aggrieved can, we suppose, make a defence, so that both sides can be heard from. The *American Convention* appears to have enemies in its midst, and its struggle for existence has been a long and severe one. This society has been patronized by a few New York dentists, since the organization of the "American Dental Asoociation" which absorbed all of its leading members, and lately another rival, under the name of the "American Academy of Dental Science," has been instituted in New York city, which bids fair to completely annihilate the "American Convention." The following is the article referred to:

(*Correspondence of the N. Y. Evening Express.*)

LONG BRANCH, August 11, 1875.

"The Dental Convention, which has been in session here for two days, and which closes to-morrow, has been marked by some peculiar proceedings. A certain defunct dentist of the city of New York, in order to gain a new base in professional life, read a poem. This gentleman continually interrupted the President of the Convention in his honest efforts so much that the latter gentleman has put in no appearance to-day.

The Dentist heretofore referred to, although known to be a man of no literary attainment, not knowing even how to speak a sentence grammatically, fostered upon the convention a poem supposed to be his own, although members of the Convention, who knew him, were well aware that it was not his own composition. It was plain to all present, that this gentleman wished to carry on the Convention for one purpose, that is an advertisement of himself. However, there was material in the Convention, which they will now carry home in their pockets, that would have enhanced the interest of the dental profession had they even but given the opportunity to do so; matters of importance, to the country, as well as the profession, would have been introduced, had it not shown itself in the Convention, that jealousy prevailed among certain parties against certain New York dentists, who had the advancement and interest of the profession at heart, the subject matter of which I allude to, is held for the third Tuesday in October, when the American Academy of Dental Surgery convenes in New York, to wit.: 'the appointment of a Dental Surgeon to the Military Academy of West Point and the School of Annapolis, also to different corps of the U. S. Army, which has been under consideration of the Government since 1869.' A paper then will be read setting forth what has been done, and the advancement made in that direction.

The gentleman heretofore mentioned, for the consideration of $10, (which fact he acknowledged in public to the members of the Convention,) bought up the little local sheet and placed two colums of solid matter at his service, in which he eulogises himself in a self-praising manner, to say nothing of publishing his beautiful poem on Grant and his prancing steeds, and high life at Long Branch; this poem, which it can be unmistakably seen, was written by a lady, took the space of over a column, while his speech on 'the great President,' as he dubs Grant, was handed to the local sheet for publication early Monday morning, long before the Convention went into session.

The 21st annual meeting of the American Dental Convention has come to a conclusion without any apparent professional success. It was, however, shortly urged by a member from New York to bring the young men to the front and lift the Convention from the rut, in which it has been for the last four years or more. Had the Convention been managed in a more thorough aud business manner, Capt. Leland, who placed the Ocean Hotel at their disposal, would have been honored by over 500 of the dental fraternity, instead of the small number who felt interested enough to attend."

